# The endocanabinnoid system and diabetes - critical analyses of studies conducted with rimonabant

**DOI:** 10.1186/1758-5996-1-18

**Published:** 2009-10-16

**Authors:** Ada Letícia B Murro

**Affiliations:** 1 Disciplina de Endocrinologia, Departamento de Clínica Médica, Universidade Estadual de Campinas, Campinas, SP, Brazil; 2 Disciplina de Endocrinologia, Faculdade de Ciências Médicas, R Vital Brazil, 250, Cidade Universitária Zeferino Vaz, Barão Geraldo, CEP: 13083990, Campinas-SP, Brazil

## Abstract

Rimonabant is the first CB1 receptor inhibitor available in the Brazilian market. This new drug has been approved for the treatment of obese or overweight patients associated with cardiovascular risk factors. In this article it is compared the effects of rimonabant treatment in obese patients with cardiovascular risk factors to usual obesity pharmacological treatment.

## Background

Rimonabant is the first CB1 receptor inhibitor available in the Brazilian market. This new drug has been approved for the treatment of obese or overweight patients associated with cardiovascular risk factors. This way, it is important to emphasize that the Rimonabant drug has not been approved for the treatment of patients with isolated cardiovascular risks, but rather for the treatment of excess weight. It is questioned whether the effect of Rimonabant regarding cardiovascular risk factors (lowering blood pressure, promoting lipid profile improvement, and reduction of glycated hemoglobin) is related to weight loss, or whether risk factor improvement is superior to other anti-obesity medications or to weight loss based on life style change.

Studies show that Rimonabant-driven weight loss is superior compared to placebo. However, to date, there are no studies which compare Rimonabant to other anti-obesity drugs or intensive life style change, such as the Diabetes Prevention Programme (DPP). The DPP compared the effects of the use of metformin to the change in life style and it was shown that the latter resulted in greater weight loss and cardiovascular risk factor improvement.

Next, the effects of Rimonabant over each cardiovascular risk will be discussed.

## Blood Pressure

Rimonabant has been announced as the drug to result in blood pressure reduction. The study to show the most relevant blood pressure drop was the RIO-Lipids, which evinced a 2.1-mmHg in systolic BP in the Rimonabant group, and a 1.7-mmHg drop upon the subtraction of the placebo effect [1]. Although statistically relevant, the little clinical significance of this value leads to the conclusion that the effect of this drug on blood pressure is null.

## Lipids

Regarding lipids in multivariate analyses, there seems to be a non-weight-dependent percentage of improvement. The use of Rimonabant compared to placebo resulted in a difference of nearly 12% in HDL-cholesterol increase and a nearly 12% increase in triglycerides levels. The presentation of the effect of the drug over a certain variable through a percentage value, although useful from the statistical standpoint, might many times overshadow the magnitude of the actual effect possibly expected from the drug, in absolute numbers. Therefore, it is worth observing the effect caused by Rimonabant, on average, in absolute numbers over lipids. In the RIO-Lipids study, the increase in HDL-cholesterol was 3.13 mg/dL greater than the placebo, on average, on the Rimonabant group[1]. The RIO-Diabetes study shows, as a result, an average improvement of 3.9 mg/dL in men upon the subtraction of the placebo effect[2]. The residual impact of Rimonabant over HDL, i.e., the post-adjustment effect for weight loss, was accountable for 57% of the improvement. Again, analyzing in absolute numbers, it was found that Rimonabant, regardless of the weight loss, led to an average 2.22 mg/dL increase in HDL. Analyzing the group that presented weight loss under 5% of the initial weight, the Rimonabant group presented a 1.66% increase in HDL, on average. The issue brought about when assessing the effect of the drug, regardless of the weight, is whether the improvement is clinically significant and if it can be converted into genuine reduction in cardiovascular risk.

Comparing Rimonabant to other drugs which also promote weight loss like sibutramine (an anti-obesity drug, whose effect is similar to Rimonabant, except for its non-effect over the endocanabinnoid system), within a year, it was observed that weight loss was accompanied by a 15% increase in HDL cholesterol[3]. This improvement is due to weight loss, once this is the only effect of the drug.

Among patients who lost less than 5% of their weight, the Rimonabant group presented a 5.2% reduction in triglycerides, on average, compared to placebo. Once again, the question is raised as to the clinical meaning of this reduction.

These data lead us to question if the weight loss induced by Rimonabant brings any additional clinically significant benefit when compared to weight loss by any other anti-obesity drug, or even due to changes in life style.

Studies published to date do not demonstrate an improvement in LDL, which is considered the cholesterol fraction to be more closely linked to cardiovascular risk.

## Glycemic control

The two studies available and published with Rimonabant in diabetic patients, Rio-Diabetes and Serenade, showed an improvement in glycated hemoglobin of nearly 0.5% compared to placebo[4].

Anderson et col. published in 2003 a meta-analysis of various studies which interfered in diabetic patients' weight[5]. It demonstrated that, regarding baseline values, there was an improvement in several parameters such as the reduction of fasting glycemia, cholesterol, LDL, triglycerides and systolic and diastolic blood pressure. The only factor where an improvement was not observed was in HDL, although some studies revealed a worsening of the condition. The conclusion of the meta-analysis was that weight reduction in diabetic patients, regardless of the treatment, improves cardiovascular risk, except HDL, in a comparable magnitude to that observed with Rimonabant. In this meta-analysis, no mentions were made to glycated hemoglobin. In a meta-analysis of studies conducted with sibutramine, considering glycated hemoglobin as a parameter, a substantial variability was observed in the decrease of HbA1c (0 to 1.34%), with an average reduction of 0.28%. Of the studies included, only three presented statistical significance[6]. Considering these data, it is possible that Rimonabant is more effective in reducing glycated hemoglobin than weight loss alone. However, the change in stratified HbA1c by weight loss was not published, which would lead us to conclude if there is actually an improvement beyond weight loss. Thus, the doubt remains whether the 0.5% reduction in glycated hemoglobin is weight-dependent or if this reduction goes beyond weight loss.

## Inflammatory Markers

Regarding inflammatory markers, which are currently also considered cardiovascular risk markers, their improvement was also observed in studies with Rimonabant. In the RIO-Lipids study, there was a 29% reduction in the C reactive protein (CRP), and in the RIO-Diabetes study, the CRP reduction was 26% [2]. Recently, Copolla et cols. demonstrated that the reduction of BMI from 34.4 to 30.1 on average with a multidisciplinary treatment in the course of one year was followed by a 40% drop in CRP levels[7], i.e., effective weight loss may improve CRP levels in greater magnitude than with Rimonabant.

Considering adiponectin, the RIO-Lipids study showed an increase in adiponectin to 1.5 μg/ml, upon subtraction of the placebo effect. A multivariate analysis demonstrated that 57% of this effect would be a result of the blockage of the CB1 receptor rather than weight loss. However, the same study already previously mentioned, which demonstrated the reduction of CRP with weight loss, also evaluated adiponectin, having observed an average increase of 58.8% in adiponectin[7].

Also, despite the low levels of adiponectin having been related to greater cardiovascular risk, it is worth pointing out that the clinical significance of adiponectin increase is yet undefined. Glitazones, sensitizers of the insulin action which increase adiponectin levels more remarkably than Rimonabant[8], still have not proven to reduce cardiovascular risk.

It is known that other drugs, such as glimepiride, are capable of increasing adiponectin levels up to 50%, despite their lack of effect over body weight[9].

Another factor that results in an increase in adiponectin levels is physical exercise, possibly due to an improvement in insulin resistance. In a study where physical exercise was performed through a period of 10 weeks, without leading to weight loss, the average adiponectin level increased over 100%[10]. These data suggest that the observed adiponectin increase with Rimonabant is inferior to that obtained with the above described interventions. Also, once again, the significance of this increase is still undefined.

Another factor worth emphasizing is the way this datum is presented, both in publications and in product promotional materials - at times, it conveys the impression that the magnitude of the event is greater than what is verified in absolute numbers. As an example, the information that 45% of the improvement in HDL observed in studies with Rimonabant is not explained by weight loss sounds more important than the information that a 3.6% increase in HDL induced by Rimonabant is not explained by weight loss, specially considering that this increase would be nearly 1.4 mg/dL in absolute numbers. Also, and once again, the clinical significance of this change is still questioned.

## Atheromatous plaque

The Stradivarius study recently published, analyzed the effect of the use of Rimonabant over the atheromatous plaque, which was being expected as it was the first study to propose to assess if the use of Rimonabant did actually result in the reduction of the atheromatous plaque evaluated by the coronary ultrasound. In this study, according to the primary endpoint, the percentage volume of the atheromatous plaque did not show an improvement compared to placebo. The only significant improvement among Rimonabant users compared to placebo was observed in the subgroup that was not given statins[11]. Considering that most patients to whom Rimonabant is indicated have high cardiovascular risk, these patients must already be using statins, i.e., this subgroup probably corresponds to a small share of the high cardiovascular risk population.

## Safety

A meta-analysis of the safety and efficacy of Rimonabant was recently published, evaluating the data of four studies with Rimonabant, at a total of 4105 patients. The average weight loss was 4.7 kg, having subtracted the placebo effect; patients using Rimonabant had a chance five times greater of presenting weight loss over 2%. The main adverse effects reported, which in reality is the greatest concern of us physicians, were of psychiatric nature[12]. One of endocannabinnoid agonists is known as anandamide. The term ananda, from Sanskrit, means "internal bliss". The term might reveal well some of the physiological functions of the endocannabinoid system in the central nervous system. Di Marzo summarized some of these functions as "feeling less pain, controlling one's movements, relaxing, eating, forgetting, sleeping and protecting"[13]. This way, it is expected that the intervention in this system be followed by other effects rather than the reduction in food intake.

Objectively, the relative risk of a patient using Rimonabant developing an adverse event is 1.4, and the NNH *(number needed to harm)*, i.e., the number of patients treated so that one patient will present any adverse event, is 25. For a serious adverse event, the NNH is 59. The relative risk of a patient taking Rimonabant to discontinue the medication due to depression is 2.5. For every 49 patients treated, one discontinued the treatment due to depression, and for every 166 patients treated, one had to stop due to anxiety[12].

## Final considerations

It is undebatable that weight loss induced by Rimonabant is clinically significant and that it results in a metabolic profile. However, weight loss cannot be mistaken for other more clinically relevant endpoints, and the greatest concern and expectation of the medical community revolves around cardiovascular risk, whose reduction still has not been demonstrated. The termination of other ongoing studies, the main being the CRESCENDO study, will show if the drug genuinely brings effect to cardiovascular mortality, which in the end is what matters the most to us, and clarify if this effect adds to that of drugs which present a well established cardiovascular benefit, such as statins.

## Competing interests

The author declares that she has no competing interests.
